# Exploring the neuroprotective activity of a lignanamides-rich extract in human neuroblastoma SH-SY5Y cells under dimethyl sulfoxide-induced stress

**DOI:** 10.3389/fcell.2024.1374626

**Published:** 2024-03-13

**Authors:** Marta Mallardo, Severina Pacifico, Simona Piccolella, Irene Di Meo, Maria Rosaria Rizzo, Aurora Daniele, Ersilia Nigro

**Affiliations:** ^1^ Dipartimento Di Scienze e Tecnologie Ambientali, Biologiche, Farmaceutiche, Università Della Campania “Luigi Vanvitelli”, Caserta, Italy; ^2^ CEINGE, Biotecnologie Avanzate Scarl, Naples, Italy; ^3^ Dipartimento di Scienze Mediche e Chirurgiche Avanzate, Università Della Campania “Luigi Vanvitelli”, Napoli, Italy; ^4^ Dipartimento di Medicina Molecolare e Biotecnologie Mediche, Università Degli Studi di Napoli “Federico II”, Naples, Italy

**Keywords:** neurotoxicity, inflammation, oxidative stress, neuronal and glial cells, dimethyl sulfoxide, phenylamides and lignanamides

## Abstract

**Introduction:** Dimethyl sulfoxide (DMSO) is widely used as a diluent and/or solvent for pharmacological compounds. Furthermore, DMSO crosses the blood-brain barrier acting on the nervous system. The natural compounds phenylamides and lignanamides (LnHS) have protective effects on neuronal health, being promising neuroprotective candidates. In this scenario, we evaluated the impact of DMSO and/or LnHS on SH-SY5Y and U-87 cells, taken as *in vitro* model of neurons and glia.

**Methods:** Cells were treated with DMSO and/or LnHS at different doses and proliferation (MTT and trypan blue counting, colony forming ability, autophagy, oxidative stress (NO, ROS determination) and inflammatory (IL8, IL6, TNFα mRNA expression) response was evaluated.

**Results:** We found that DMSO reduces both neuronal and glial cell viability, while LnHS does not affect viability of SH-SY5Y cells but reduces that of U-87 cells. Therefore, we focused on SHSY5Y cells and investigated whether LnHS could counteract DMSO toxicity. LnHS partially attenuates the inhibitory effects of DMSO on cell viability and restores the colony-forming ability of SH-SY5Y cells exposed to DMSO. Furthermore, we found that co-administration of LnHS modulates the expression of SIRT3 and SOD2 enzymes, reduces nitrite release and ROS generation increasing IL-8 levels. Interestingly, co-administration of LnHS counteracts the DMSO-induced production of IL-6, while no modification in TNF-α was found.

**Discussion:** Our study indicates LnHS as a potential feasible compound to support neuronal health as it counteracts DMSO induced cytotoxic effects by improving SH-SY5Y cells survival. Further studies are needed to clarify the molecular mechanisms underlying the LnHS biological activities.

## Graphical Abstract

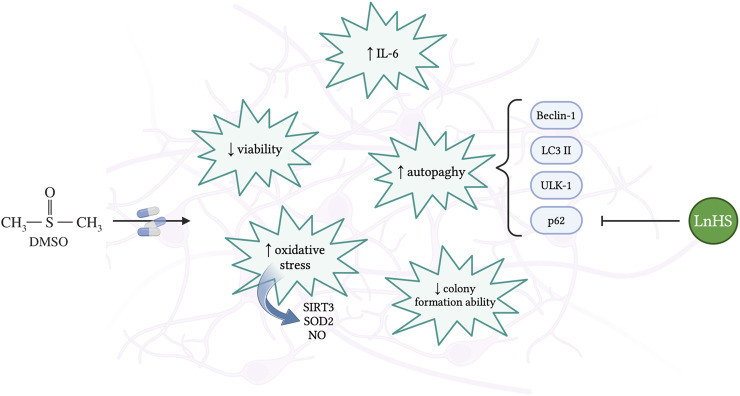

## 1 Introduction

Dimethyl sulfoxide (DMSO) is a widely used polar organic solvent with various biological applications (Wojaczynska and Wojaczynski, 2023). In the field of neuroscience, DMSO allows the administration of various pharmacological compounds and is therefore widely used as a vehicle for drug therapy (Santos et al., 2003). It is in fact a non-enzymatic antioxidant compound that interacts with the hydroxyl group present on any substance and is able to cross the organic membranes bringing with it other substances to which it joins (Gironi et al., 2020). Furthermore, DMSO is widely used in vitro and *in vivo* studies because, by facilitating the administration of substances insoluble in water, it acts as a vehicle for testing various pharmacological therapies (Blevins et al., 2002). However, numerous studies suggest that DMSO may have toxic effects or induce nonspecific outcomes (Blevins et al., 2002; Hanslick et al., 2009; Gironi et al., 2020). It has been described that the peripheral injection of DMSO produces toxic and/or adverse effects, while less is known about whether DMSO produces toxic effects following central injections (Blevins et al., 2002). In fact, peripheral injections of DMSO have been shown to elicit changes in tissue, proteins, DNA, and enzymes (Friedman, 1967; Highman and Altland, 1967; Solveig and Erixon, 1984; Iwatani et al., 2006). Only one study on vehicle effects following central administration reported that DMSO (10%) reduced exploratory behavior compared to other vehicles (saline, Tween-80 (2%), or propylene glycol (10%)) in mouse models (Matheus et al., 1997). Nevertheless, although conflicting results are reported in the literature regarding the effects of DMSO on cells of the nervous system, most of data suggest that DMSO exerts neurological side effects and toxicity (Hanslick et al., 2009; Wojaczynska and Wojaczynski, 2023). Regarding the molecular effects induced by DMSO, few studies analyzed such mechanisms in cellular models suggesting that this molecule might be implicated in alterations of essential cellular components, such as proteins and DNA (Tuncer et al., 2018). Furthermore, DMSO can induce adverse effects in human cellular models including generation of oxidative stress and inflammatory mediators (Verheijen et al., 2019). In particular, it has been demonstrated that IL-8 has an interesting relationship with oxidative reaction since the gene expression of this cytokine is highly sensitive to oxidants (DeForge et al., 1993). In addition to oxidative stress, mitochondrial malfunction seems also to be implicated in DMSO detrimental effects; sirtuins are the main regulators of mitochondrial stress response (Yuan et al., 2014). The sirtuin family comprises a group of highly conserved nicotinamide adenine dinucleotide (NAD+)-dependent protein deacetylases, consisting of seven species in mammals, known as sirtuin 1–7 (SIRT1-7) (Wang et al., 2022).

Hemp seeds (HS) are actually of great interest, because of its richness in n-3 and n-6 essential fatty acids, mainly linoleic acid and α-linolenic acid, phytosterols, phenolamides and other antioxidant polyphenols (Nigro et al., 2020; Di Palo et al., 2022; Nigro et al., 2022). Phenylamides and lignanamides constitute a small group of natural compounds with their broader distribution in the plant kingdom (Nigro et al., 2020). These compounds have been found to display interesting and diverse biological activities, including anti-inflammatory activities which suggest the consumption of HS for the prevention of several pathological conditions such as cardiovascular disorders (Nigro et al., 2020; Xu et al., 2023). Nevertheless, one of the most crucial features of lignanamides is the ability to preserve the neuronal health, making them promising neuroprotective candidates. Furthermore, N-trans-caffeoyltyramine, and cannabisin B, the main Hemp seed (HS) Lignanamides Rich-Fraction (LnHS) constituents, have protective effects on the central nervous system (Wang et al., 2019; Nigro et al., 2020). The same extract modifies the miRNome of cultured human neural cells with effects on specific microRNAs involved in neural functions (Di Palo et al., 2022).

Considering that DMSO neurotoxic effects were previously evidenced and that LnHS has been found to have beneficial effects, the aim of the present study was to investigate the effect of DMSO and/or LnHS and of a combined treatment on SH-SY5Y and U-87 cells, used as an *in vitro* model of neurons and glial cells respectively. In particular, we evaluated whether LnHS, specifically on SHSY5Y, could mitigate the detrimental effects induced by DMSO in terms of cell viability, clonogenic ability and autophagic mechanisms. Furthermore, we investigated the potential ability of LnHS to alleviate DMSO-induced oxidative stress by evaluating intracellular production of ROS, the expression of SIRT3 and SOD2 enzymes, along with nitrite release and IL-8 mRNA levels. Finally, we evaluated LnHS effects on the expression levels of pro-inflammatory cytokines such as IL-6 and TNF-α.

## 2 Materials and methods

### 2.1 LnHS extract obtainment and its chemical profiling

LnHS extract was obtained from cryo-crushed and defatted HS commercially obtained (Hanf & Natur product, Lindlar, Germany), previously extracted with n-hexane, underwent ultrasound-assisted maceration (UAM) by using an HS/ethanol ratio equal to 1 g: 5 mL. The ethanol extract was further fractionated by SiO_2_ column chromatography eluting with solvents at increasing polarity (chloroform, ethyl acetate, and methanol). Thus, the most polar fraction was chemically investigated through UHPLC-HRMS and HPLC-UV-DAD analyses.

The employed equipment and parameters have been previously detailed (16). Briefly, to achieve the best compound resolution in liquid chromatography an elution gradient was optimized on a Luna^®^ Omega Polar C18 column (1.6 μm particle size, 50 × 2.1 mm, Phenomenex, Torrance, CA, United States), as reported in Table 1.

**TABLE 1:** LC elution gradient optimized for LnHS (A **=** H_2_O (0.1% HCOOH); B = CH_3_CN (0.1% HCOOH).

Time (min)	A (%)	B (%)
0.0	98	2
3.0	87.5	12.5
12.5	70	30
17.5	55	45
20.0	25	75
22.0	25	75
23.0	2	98
24.0	2	98

2 min re-equilibration time.

HRMS information was obtained by using the AB SCIEX Triple TOF^®^ 4600 (AB Sciex, Concord, ON, Canada) coupled with the NEXERA UHPLC (Shimadzu, Tokyo, Japan), applying the following potentials (V): ion spray voltage (−4500), declustering potential (−80), collision energy (−45 ± 15). The other parameters regarding the MS ion source were: curtain gas (35 psi), nebulizer/heater gases (60/60 psi), and interface heater temperature (600°C).

DAD-UV-Vis data were gained by the Agilent WR G7115A diode array detector of the 1260 Infinity II LC system (Agilent, Santa Clara, CA, United States).

### 2.2 Cell-based assays

#### 2.2.1 Cell cultures

The human neuroblastoma (SH-SY5Y) and glioblastoma (U-87) cell lines were kindly provided by the Bank of Human and Animal Continuous Cell Lines-CEINGE Biotecnologie Avanzate “Franco Salvatore”, Napoli, Italy. The SHSY5Y cell line was used for the experiments in its undifferentiated form. Both cell lines were cultured in Dulbecco’s medium (DMEM) (Sigma-Aldrich, MO, United States) supplemented with 10% of heat-inactivated Fetal Bovine Serum (FBS) (Sigma-Aldrich, MO, United States) and 1% l-glutamine (Sigma-Aldrich, MO, USA). Cells were grown in a 5% CO2 humidified incubator, at 37°C. To choose the optimal doses for the experiments, dose-response curves were performed for both DMSO (Sigma-Aldrich, MO, USA) and LnHS by treating the cells with increasing concentrations of the compounds (1%, 2.5%, 5%, and 10% v/v for DMSO; 2.5, 5, and 10 μg/mL for LnHS). All experiments were performed in 2% FBS medium, except for the colony forming assay, which was performed in 5% FBS medium.

#### 2.2.2 Cell viability assays

Cell viability was assessed using the 3-[4,5-dimethylthiazol-2-yl]-2,5-diphenyltetrazolium bromide (MTT) colorimetric assay as previously reported (Nigro et al., 2021; Nigro et al., 2022). Briefly, SH-SY5Y (4×10^3^ cells/well) and U-87 (2×10^3^ cells/well) were seeded in 96-well plates. The following day, the cells were treated with increasing concentrations of DMSO (1%, 2.5%, 5%, and 10% v/v) (Sigma-Aldrich, MO, USA), or treated with LnHS (0.5, 2.5, 5, and 10 μg/mL), or co-treated with 1% DMSO and LnHS (2.5, 5, and 10 μg/mL). Cells incubated in medium alone served as the control. After 24, 48, and 72 h of treatment, cells were stained with 5 mg/mL of MTT reagent for approximately 4 h. The formazan crystals that were formed were further dissolved in 100 μL of DMSO and the absorbance was measured at 550 nm using a microplate reader (Model 550, Ultramar Microplate Reader; Bio-Rad, CA, United States). The final readings were compared with the untreated control samples. The experiments were performed twice in triplicate.

Additionally, cell viability was also measured using the trypan blue reagent (Bio-Rad, CA, USA). Briefly, SH-SY5Y (1 × 10^4^ cells/well) were seeded in 24-well plates. The following day, cells were treated with 1% DMSO or co-treated with 1% DMSO and LnHS (2.5, 5, and 10 μg/mL). After 24, 48, and 72 h, cells were harvested, stained for 5 min with 0.4% trypan blue according to the manufacturer’s instructions, and counted using the TC10TM Automated Cell Counter (Bio-Rad, CA, United Staes). The experiments were performed twice in triplicate.

#### 2.2.3 Colony forming assay

SH-SY5Y cells were seeded at a density of 1×10^3^ cells per well in 6-well plates and incubated overnight. The following day, the cells were treated with 1% DMSO, or various concentrations of LnHS (2.5, 5, and 10 μg/mL), or co-treated with 1% DMSO and LnHS (5 and 10 μg/mL) in culture media containing 5% FBS. The growth medium was replenished every 3 days. After a 14-day incubation period, the plates were washed twice with PBS, fixed with 4% paraformaldehyde (PFA) (Invitrogen, CA, United States) for 30 min, stained with a 1% crystal violet solution (Sigma-Aldrich, MO, United States) for 20 min, and then washed repeatedly with water. Colonies were manually counted using a light microscope as previously described. Each experiment was performed twice in triplicate.

#### 2.2.4 Western blotting

Total protein content was extracted from SH-SY5Y cells using pre-cooled radioimmunoprecipitation assay (RIPA) buffer (Sigma-Aldrich, MO, United States) containing a protease inhibitor cocktail (Abcam, Cambridge, UK). Subsequently, the samples were centrifuged at 12.500 rpm for 15 min at 4°C. The supernatant was collected, and the protein content was quantified using the Bradford method (Bio-Rad, CA, United States). Then, the samples were diluted in 4X Laemmli buffer and boiled for 5 min at 95°C.

Typically, 25 μg of total cellular proteins were loaded onto a polyacrylamide gel (10%) and separated by SDS-PAGE. The proteins were then transferred to PVDF membranes (Pierce Biotechnology, Massachusetts, United States). To prevent non-specific binding of detection antibodies during the steps following transfer, unoccupied sites on the membrane surface were blocked with a 5% non-fat milk (TBST) solution for 1 h. Subsequently, the membranes were incubated overnight at 4°C with the following primary antibodies according to the manufacturer’s instructions: rabbit monoclonal anti-ULK-1 (1:1000), rabbit monoclonal anti-p62 (1:1000), mouse monoclonal anti-Beclin-1 (1:1000), rabbit monoclonal anti-LC3A-B (1:1000), and mouse monoclonal anti-GAPDH (1:3000) (Cell Signaling Technology, Massachusetts, United States). The next day, the membranes were incubated with anti-rabbit (1:3000) and anti-mouse (1:3000) secondary antibodies coupled to horseradish peroxidase (Cell Signaling Technology, Massachusetts, United States). The membranes were washed three times with TBS Tween-1X (Thermo Fisher Scientific, Massachusetts, United States) before and after each incubation step. Finally, the protein bands were detected using a Chemi Doc XRS system (Bio-Rad, CA, United States) with ECL detection reagents (Elabscience, Texas, United States). The experiments were performed twice in triplicate.

#### 2.2.5 RNA extraction and quantitative real time-PCR

Total RNA was extracted from SH-SY5Y cells by using TRIzol Reagent (Thermo Fischer Scientific, Massachusetts, United States). The RNA concentration was quantified by using fluorescence-based detection with Qubit 4 Fluorometer (Thermo Fischer Scientific, Massachusetts, United States). cDNA was synthesized using 1000 ng total RNA with SuperScript IV VILO Master Mix (Thermo Fischer Scientific, Massachusetts, United States) according to the manufacturer’s instructions. Gene expression was performed in C1000 Touch Thermal Cycler (Bio-Rad) using iQ SYBR Green Supermix (Bio-Rad) with the following thermal cycling parameters: 95°C, 3 min, followed by 40 cycles of denaturation (95°C, 10 s), annealing (60°C, 30 s), and elongation (72°C, 30 s). GAPDH was used as housekeeping gene; fold changes were calculated with the 2^−ΔΔCT^ method. The experiments were performed two times in duplicate. Primer sequences and their annealing temperature used for q-RT-PCR are shown in Table 2.

**TABLE 2:** Primer sequences used for q-RT-PCR experiments.

Target gene	Primer sequence (5′-3′)	Primer annealing temperature (°C)
SOD 2	F: CCT​ACG​TGA​ACA​ACC​TGA​AC	51.8
	R: GAA​GAG​CTA​TCT​GGG​CTG​TA	51.8
SIRT 3	F: CCTTGGCTTGGCATCCTC	52.6
	R: GCA​CAA​GGT​CCC​GCA​TCT​C	55.4
IL-8	F: GGA​GAA​GTT​TTT​GAA​GAG​GGC​TGA	55.7
	R: GAA​TCT​TGT​ATT​GCA​TCT​GGC​AAC	54.0
IL-6	F: GAT​GAG​TAC​AAA​GTC​CTG​ATC​CA	59.0
	R: CTG​CAG​CCA​CTG​GTT​CTG​T	60.0
TNF-α	F: AGC​CCA​TGT​TGT​AGC​AAA​CC	51.8
	R: TGA​GGT​ACA​GGC​CCT​CTG​AT	53.8
GAPDH	F: CAT​GGC​CTT​CCG​TGT​TCC​TA	54.0
	R: CCT​GCT​TCA​CCA​CCT​TCT​TGA​T	54.8

#### 2.2.6 NO determination

The release of nitric oxide (NO) in the culture media was assessed using the Griess reagent, following the manufacturer’s instructions. Briefly, SH-SY5Y (4×10^3^ cells/well) were plated in 6-well plates and incubated overnight. The following day, the cells were treated with 1% DMSO or with LnHS (5 and 10 μg/mL), or co-treated with 1% DMSO and LnHS (5 and 10 μg/mL) for 48 h. The next day, 100 μL of supernatants were mixed with 50 μL of Griess reagent solution A (1% sulfanilamide w/v in 10% HCl 37% v/v) for 5 min, followed by the addition of 50 μL of Griess reagent solution B (0.1% N-(1-naphthyl) ethylenediaminedihydrochloride). After incubating for 10 min at RT, the absorbance was measured at 540 nm and compared to the absorbance of NaNO2 standard solutions. Each experiment was performed twice in triplicate.

#### 2.2.7 ROS generation

The 2′, 7′-dichlorodihydrofluorescein diacetate (DCF-DA) fluorescent dye was used to detect ROS generation according to the manufacturer’s instruction. In brief, SH-SY5Y (3 × 10^4^ cells/well) were seeded into a 48-well plate. The next day, cells were treated with 1% DMSO and LnHS (5 μg/mL), or co-treated with both compounds for 48 h. After the incubation period, cells were washed twice with PBS and incubated in the dark with 20 μM DCF for 45 min at 37°C. Subsequently, the cells were washed with sterile PBS and observed under a microscope. All images were acquired using identical settings. The experiments were conducted twice in triplicate.

#### 2.2.8 Statistical analysis

Data are expressed as mean of replicates ±standard deviation (SD). GraphPad Prism 6 software (GraphPad Software, California, United States) was used to carry out the analyses. Statistical comparisons between the control and treatments were performed using the one-way or two-way ANOVA followed by the Tukey multiple comparisons test. A *p*-value <0.05 was considered statistically significant.

## 3 Results

### 3.1 Chemical composition of LnHS

LnHS mainly consisted of molecules belonging to phenylamide and lignanamide classes, with only a few exceptions constituted by quercetin and kaempferol pentosyl, deoxyhexosyl, and acetyl-deoxyhexosyl derivatives (Supplementary Figure S1). Among phenylamides, two octopamine and four tyramine derivatives were found, distinguishing for the hydroxycinnamoyl (*p*-coumaroyl, caffeoyl, feruloyl) moiety, together with the tri-*p*-coumaroyl derivative of spermidine (Supplementary Figure S2). Lignanamides encompassed aryl (dihydro)naphthalene-type, benzodioxanes-type, and β-arylether-type compounds, along with nor-lignanamides (Supplementary Table S1).

Their mass spectrometry and —-Vis features, whose rationalization has been pivotal for their identification, have been previously detailed (Nigro et al., 2020). As an example, as regards phenylamides, octapaminyl derivatives easily lost water, whereas neutral loss of 42, 45, and 120 Da were detected also for tyramine derivatives. Moreover, base peaks in MS/MS spectra gave information about the hydroxycinnamoyl moiety. Cannabisin A was representative of arylnaphtalene lignanamides, showing a characteristic absorbance peak in the UV spectrum at 256 nm, and a fragmentation pathway characterized by two main neutral loss, one corresponding to the tyramine unit and the other to isocyanic acid + *p*-hydroxystirene unit. Cannabisin B isomers were examples of phenyldihydronaphtalene-type structures. The loss of the cathecol unit from caffeoyl derivation as 110 Da characterized them, besides the previously mentioned rearrangement. If the molecular skeleton contained a feruloyl derivation, the loss of 124 Da was observed. Finally, phenylcoumaran-type lignandiamides (e.g., 3,3′-didemethylgrossamide) were characterized by a tyramine direct loss from the molecular ion.

### 3.2 DMSO reduces the viability of SH-SY5Y cells in a time and dose-dependent manner, while LnHS partially mitigates this effect

The potential effects of DMSO (1%, 2.5%, 5%, and 10% v/v), LnHS (0.5, 2.5, 5, and 10 μg/mL) and the combination of both (1% DMSO and 2.5, 5, and 10 μg/mL LnHS) were evaluated on of SH-SY5Y neuronal and U87 glial cells for 24, 48, and 72 h in terms of cell viability.

Regarding U87 cells, both DMSO and LnHS independently decreased the cell viability in a time- and dose-dependent manner (Supplementary Figure S3). In detail, DMSO showed toxicity at all tested doses and incubation times, while LnHS was toxic after 72 h of incubation at all doses. Such results discourage to investigate of the concomitant treatment with DMSO and LnHS in this cell model.

**FIGURE 1:**
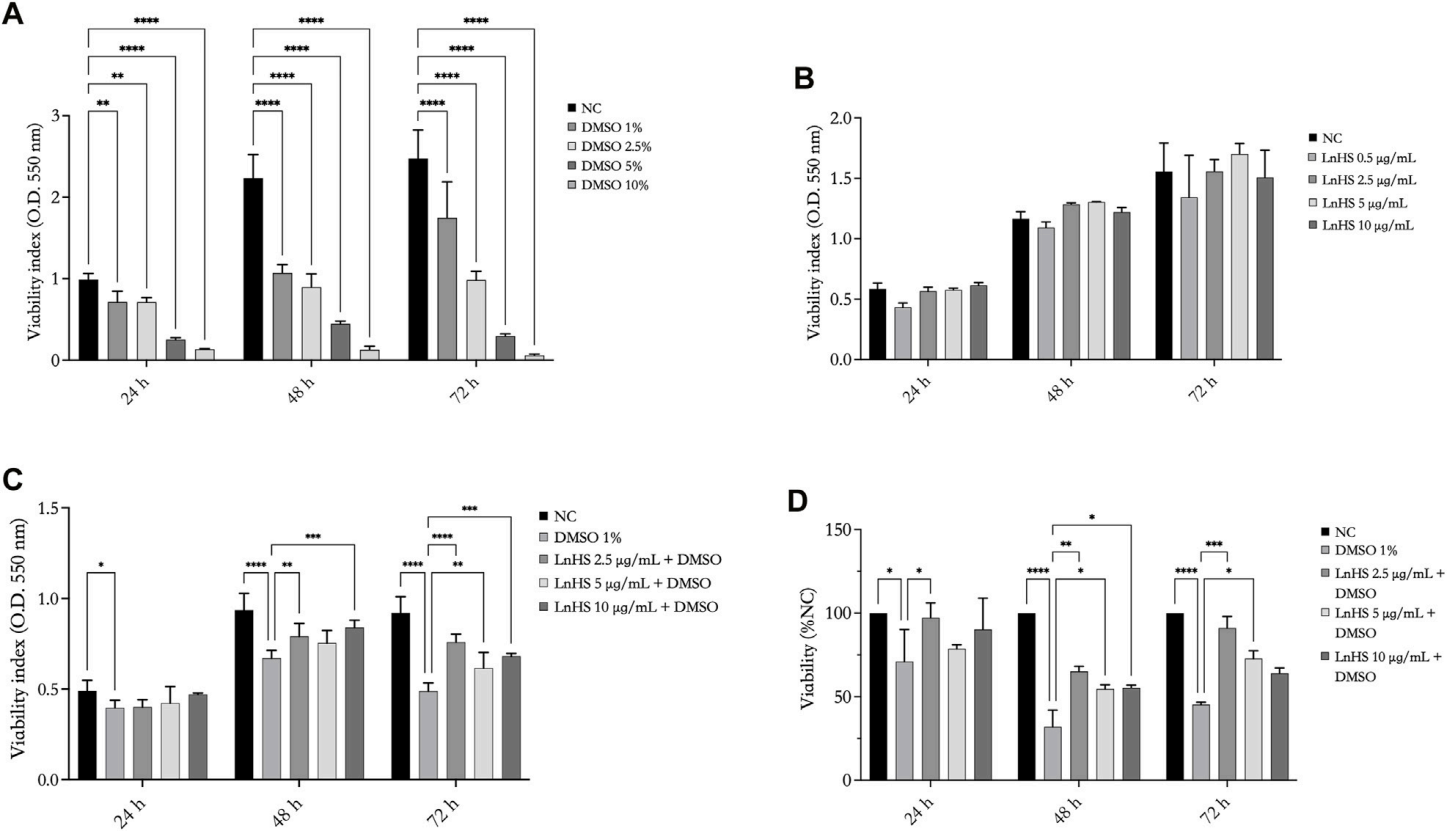
DMSO reduces the viability of SH-SY5Y cells, while LnHS partially attenuates such cytotoxic effect. Cell viability was assessed using MTT assay after exposure to **(A)** DMSO (1%, 2.5%, 5%, and 10% v/v), **(B)** LnHS (0.5, 2.5, 5, and 10 μg/mL), and **(C)** combination of both compounds (1% DMSO and 5–10 μg/mL LnHS) for 24, 48, and 72 h. **(D)** The percentage of viability was also calculated using the trypan blue assay after 24, 48 and 72 h of the treatments reported above. Values are expressed as the mean of two different experiments performed in triplicate ±SD. NC: untreated cells. The statistical analysis was evaluated using the two-way ANOVA test. *p-value <0.05; **p-value <0.01; ***p-value <0.001 ****p-value <0.0001.

As shown in Figure 1, panel A, DMSO significantly reduced the viability of SH-SY5Y cells in a time- and dose-dependent manner. Remarkably, already after 24 h, DMSO effectively reduced cell viability. Since the dose of 1% DMSO is the lowest effective concentration in reducing cell proliferation, the next experiments were performed with this dose.

To evaluate the influence of the hempseed mixture on SH-SY5Y cell viability, the cells were exposed to various concentrations of LnHS (0.5, 2.5, 5, and 10 μg/mL). As shown in Figure 1, panel B, the administration of LnHS did not impact the viability of SH-SY5Y cells at any of the tested doses and incubation times.

Therefore, our investigation focused on evaluating the potential of LnHS treatment (2.5, 5, and 10 μg/mL) in combination with DMSO (1%) to alleviate the loss of cell viability induced by DMSO on SHSY5Y cells specifically. LnHS, when used in combination with DMSO, effectively counteracts its inhibitory effects on the viability of cells at longer incubation periods (48, 72 h) (see Figure 1, panel C). A similar response was observed performing the trypan blue assay. Indeed, as shown in Figure 1, panel D, at longer incubation periods, in the combined treatment, LnHS effectively mitigates the deleterious effects on cell viability induced by DMSO.

### 3.3 DMSO significantly reduces the colony formation ability of SH-SY5Y cells, while LnHS administration effectively restores it

**FIGURE 2:**
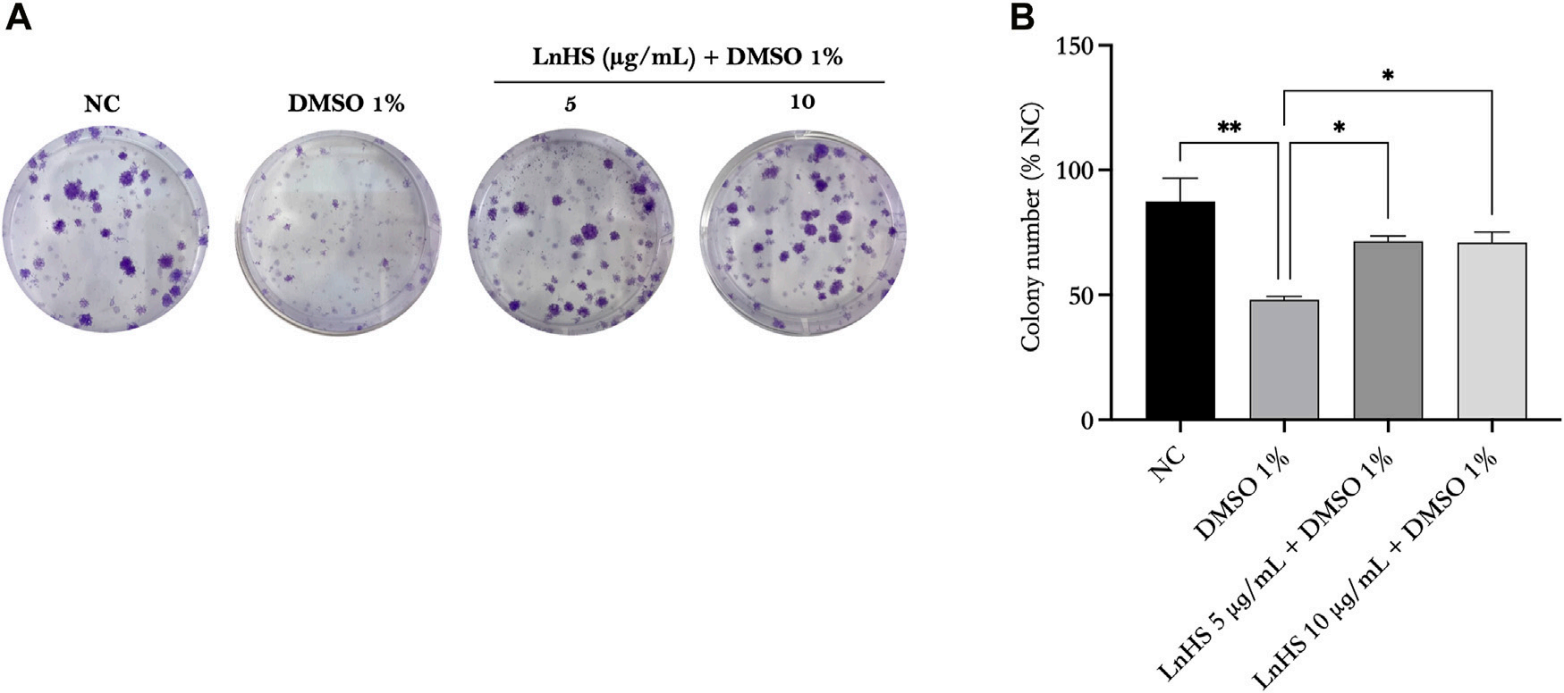
DMSO impairs colony formation in SH-SY5Y cells, while LnHS co-administration restores it. SH-SY5Y cells were exposed to 1% DMSO or a combination of 1% DMSO and LnHS (5 and 10 μg/mL) for 14 days and assessed for colony formation. **(A)** Representative images of colonies after the above-described treatment. **(B)** The number of colonies per well was counted and presented as the mean ± SD of two independent experiments performed in triplicate. NC: untreated cells. The statistical analysis was evaluated using the one-way ANOVA test. *p-value <0.05; **p-value <0.01 vs. DMSO.

Next, we evaluated the survival rate of SH-SY5Y cells after exposure to DMSO alone or in combination with LnHS via the colony forming assay. Therefore, SH-SY5Y cells were treated with 1% DMSO and co-treated with 1% DMSO and LnHS (5 and 10 μg/mL) for a duration of 14 days. As shown in Figure 2, our findings demonstrated a significant reduction in the colony forming capability of DMSO-exposed SH-SY5Y cells (48% ± 1.4%) compared to untreated cells (87.5% ± 9.1%). Interestingly, the co-treatment of LnHS with DMSO showed a remarkable ability to restore colony number (71.5% ± 2.1% for LnHS 5 μg/mL; 71% ± 4.2%).

### 3.4 LnHS attenuates DMSO-induced autophagy in SH-SY5Y cells

**FIGURE 3:**
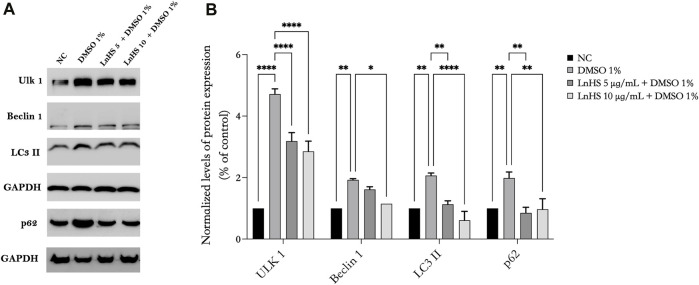
LnHS mitigates the autophagy induced by DMSO in SH-SY5Y cells. Protein extracts from SH-SY5Y cells treated with DMSO 1% or co-treated with two different doses of LnHS for 48 h were analyzed by Western blot. **(A)** A representative Western blotting image of Ulk-1, Beclin-1, LC3 II, p62 and GAPDH. **(B)** Graphical representation of pixel quantization of Ulk-1, Beclin-1, LC3 II, p62 normalized to GAPDH. Values are expressed as mean of two different experiments ±SD. NC: untreated cells. The statistical analysis was evaluated using the two-way ANOVA test. *p-value <0.05; **p-value <0.01; ***p-value <0.001; and ****p-value <0.0001 vs. DMSO.

To further explore the underlying molecular mechanisms of DMSO-induced cell toxicity and the effects of LnHS combined treatment, we analyzed, by Western blot, some key proteins involved in autophagy process. Our results demonstrated that SH-SY5Y neuronal cells exposed to 1% DMSO for 48 h exhibited significant upregulation in the expression of some autophagic proteins such as Ulk-1, Beclin-1, LC3 II, and p62 (see Figure 3). Remarkably, the co-administration of LnHS, even at a dose as low as 5 μg/mL, attenuated the upregulation effect on all tested proteins, as revealed by the Western blot analysis (see Figure 3). These findings highlight the potential efficacy of LnHS in ameliorating DMSO-induced dysregulation of autophagy in neuronal cells.

### 3.5 LnHS attenuates DMSO-induced oxidative stress in SH-SY5Y cells

**FIGURE 4:**
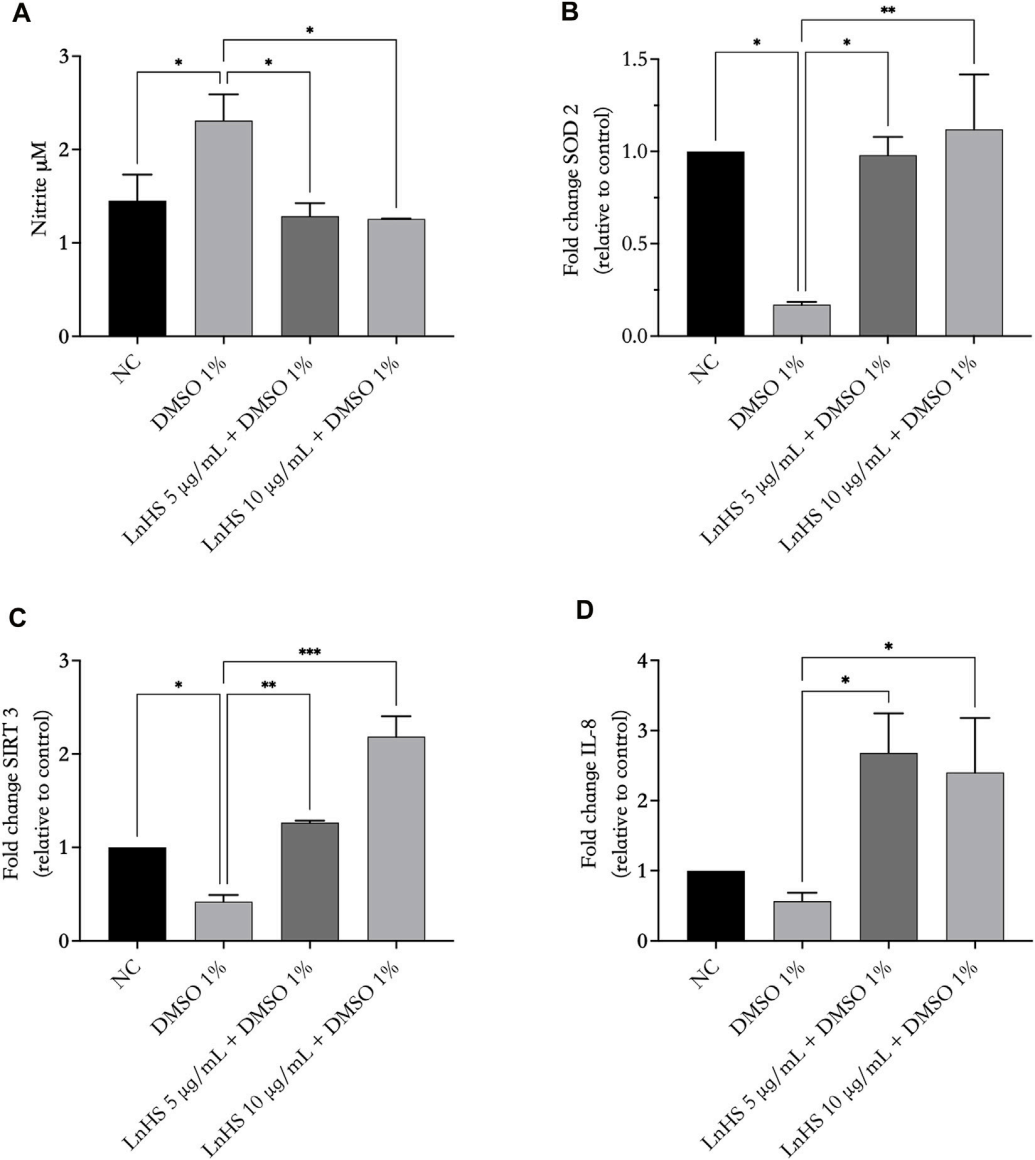
LnHS modulates DMSO-induced oxidative stress and IL-8 expression in SH-SY5Y cells. The levels of NO, SIRT3, SOD2 and IL-8 in SH-SY5Y cells were measured after 48 h of DMSO and LnHS administration. **(A)** DMSO administration significantly promoted NO production in SH-SY5Y cells, whereas LnHS significantly attenuates this effect. **(B)** DMSO significantly reduces the expression of SIRT3 and **(C)** SOD2 expression whereas LnHS restores their levels in SH-SY5Y cells. **(D)** DMSO treatment reduced IL-8 expression levels, whereas the co-administration with LnHS enhanced it. Values are expressed as the mean of two different experiments performed in triplicate ±SD. NC: untreated cells. The statistical analysis was evaluated using the one-way ANOVA test. *p-value <0.05; **p-value <0.01; ***p-value <0.001 vs. DMSO.

Next, to test the influence of DMSO and LnHS on oxidative stress, we tested NO and ROS production, as well as IL-8, SIRT3 and SOD2 expression in response to 1% DMSO alone or in combination with LnHS after 48 h of incubation. As shown in Figure 4, panel a, exposure to DMSO significantly increased the production of NO; interestingly, the combined treatment with LnHS effectively attenuated the pro-NO effects induced by DMSO. In relation to SIRT3, exposure to DMSO resulted in a significant reduction in its expression levels (see Figure 4, panel b). Notably, the combined treatment with LnHS led to an increase in SIRT3 levels (Figure 4, panel b). Furthermore, DMSO administration also significantly decreased the expression of SOD2, whereas co-treatment with LnHS resulted in increased SOD2 levels (Figure 4, panel c). Specifically, the highest dose of LnHS exhibited the greater efficacy in enhancing the levels of both antioxidant enzymes.

Due to a close relationship of IL-8 with oxidative stress, we evaluated whether the administration of 1% DMSO alone or in combination with LnHS caused potential expression changes in IL-8 at the mRNA level. As shown in Figure 4, panel d, qPCR showed that DMSO caused a reduction in IL-8 levels, although it was not significant. Interestingly, co-treatment with LnHS was effective in increasing IL-8 mRNA levels, with a similar response from LnHS 5 and 10 μg/mL treatments.

**FIGURE 5:**
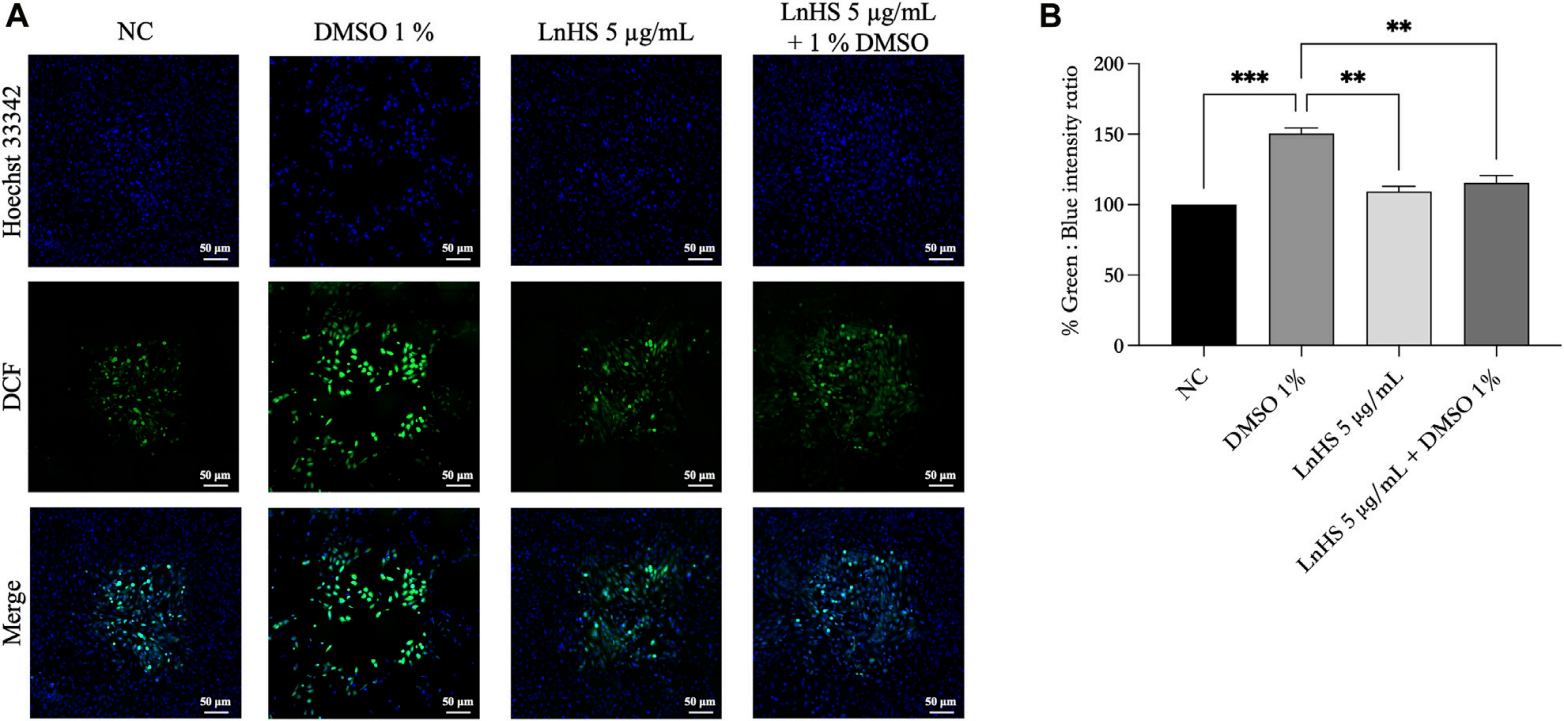
DMSO promotes ROS generation in SH-SY5Y cells, while LnHS co-administration attenuates it. **(A)** Representative fluorescence micrographs depicting ROS generation in SH-SY5Y cells after treatment with DMSO (1%) and/or LnHS (5 μg/mL) for 48 h. Scale bar is 50 μm **(B)** % of green DCFDA positive cells over total (Hoechst 33342 stained) number of cells were calculated and presented as the mean ± SD of two independent experiments performed in triplicate. NC: untreated cells. The statistical analysis was evaluated using the one-way ANOVA test. **p-value <0.01; ***p-value <0.001 vs. DMSO.

For a better understanding of the antioxidative activity of LnHS, we performed a DCFDA/H2DCFDA assay to measure intracellular ROS generation. In this experiment, SH-SY5Y cells were exposed to 1% DMSO and LnHS (5 μg/mL) or a combination of both for 48 h, followed by DCFDA incubation. As shown in Figure 5, DMSO induced an increase in intracellular ROS levels (150.5% ± 3.9%), while SH-SY5Y cells co-exposed to DMSO and LnHS exhibited a significant decrease in ROS generation (115.5% ± 5.1%), supporting the protective efficacy of LnHS against DMSO-induced ROS production. Overall, our data suggest that LnHS effectively mitigates the oxidative stress induced by DMSO.

### 3.6 LnHS alleviates DMSO-induced effects on IL-6 mRNA levels but has no impact on TNF-α levels

**FIGURE 6:**
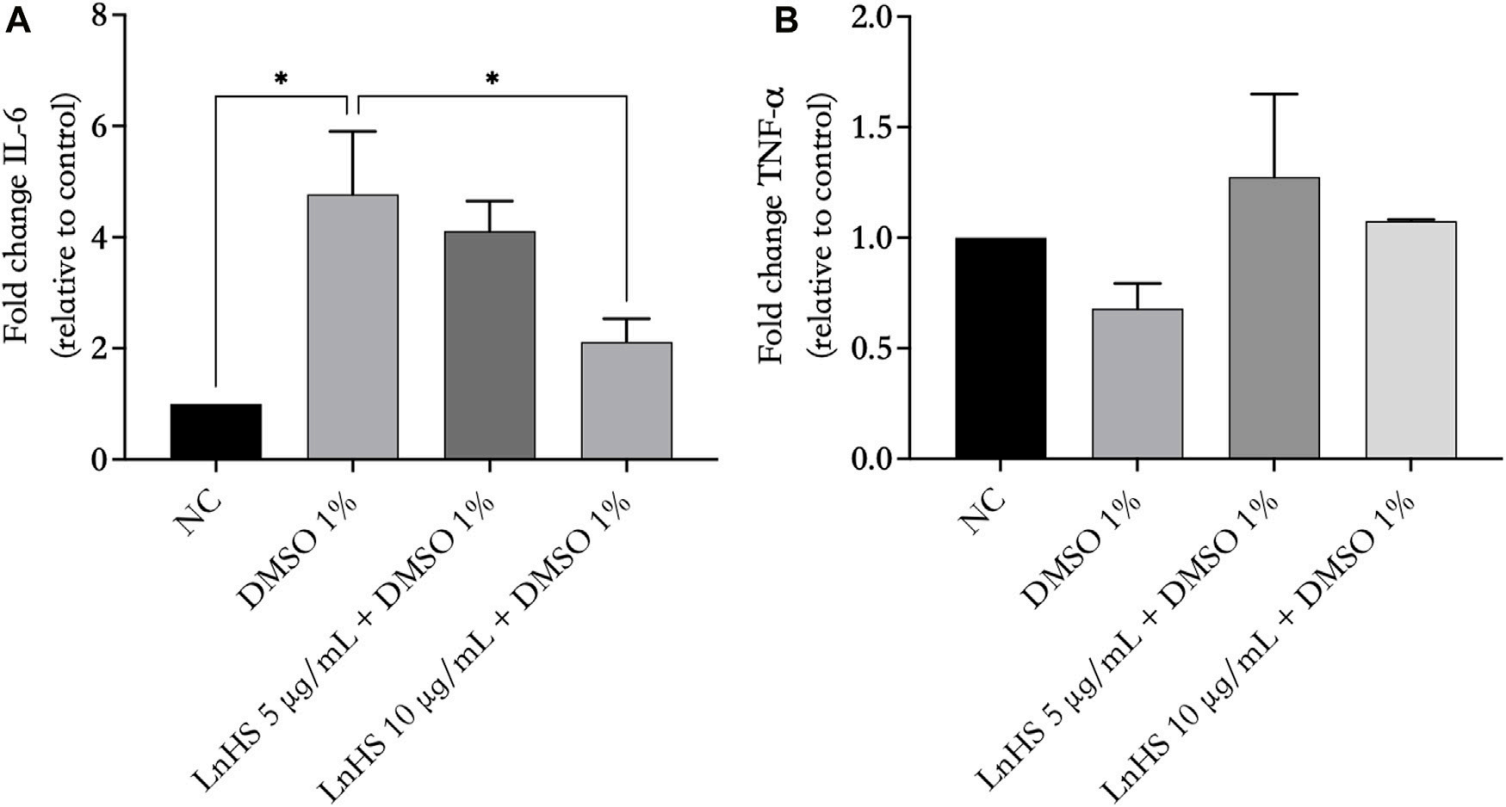
LnHS counteracts DMSO-induced effects on IL-6 mRNA levels, while neither DMSO nor LnHS modulates TNF-α mRNA levels. IL-6 and TNF-α mRNA levels were evaluated after 48 h of DMSO and LnHS administration. **(A)** DMSO administration significantly enhanced IL-6 mRNA expression levels in SH-SY5Y cells, whereas in the co-treatment LnHS attenuates this effect. **(B)** DMSO alone or in combination with LnHS did not induce any changes in TNF-α levels. Values are expressed as the mean of two different experiments performed in triplicate ±SD. NC: untreated cells. The statistical analysis was evaluated using the one-way ANOVA test. *p-value <0.05 vs. DMSO.

Next, we evaluated the ability of DMSO and LnHS co-administration to modulate the production of proinflammatory mediators such as IL-6 and TNF-α. Thus, SH-SY5Y cells were treated for 48 h with 1% DMSO alone or in combination with LnHS (5 and 10 μg/mL) to evaluate the potential expression changes in IL-6 and TNF-α at mRNA levels. As shown in Figure 5, panel a, exposure to DMSO resulted in a significant increase in pro-inflammatory IL-6 mRNA levels. Notably, the combined treatment with LnHS led to a dose-dependent decrease in IL-6. Regarding TNF-α expression, neither DMSO nor LnHS co-administration resulted in any modifications in TNF-α levels compared to control (see Figure 6, panel b).

## 4 Discussion

Currently, DMSO has important biological applications being widely used in therapeutic clinics to deliver the administration of various neuropharmaceuticals. However, DMSO has been shown to also induce neuronal injuries both in animal models and humans (Salim, 1991; Cavaletti et al., 2000; Hanslick et al., 2009; Marcacci et al., 2009).

Starting from these observations and from our previous experiences (Nigro et al., 2020), in the present study, we demonstrated that LnHS, whose neuroprotective effects are known, could counteract the toxicity induced by DMSO in SH-SY5Y cells, used as an *in vitro* model of neuronal cells. Our results revealed that LnHS administration did not significantly affect SH-SY5Y neuronal cells while exerting toxic effects towards U-87 glial cells. In combination with DMSO, LnHS improves SH-SY5Y cell viability *via* attenuation of autophagic cell death and oxidative stress, both cytotoxic effects induced by DMSO. Accordingly, we and others have previously demonstrated that LnHS influences autophagic cell death, leading to the downregulation of the autophagy process in HepG2 and U-87 cells (Chen et al., 2013; Nigro et al., 2020).

In SH-SY5Y neuronal cells, DMSO treatment increased the generation of intracellular ROS, a phenomenon that was suppressed by the co-administration of LnHS supporting the hypothesis that the protective effect of LnHS against DMSO-induced cell damage may be attributed to the inhibition of ROS generation. In accordance with our data, protective effects of some hemp components such as CBD against neuronal toxicity induced by cadmium and Aβ peptides have been previously reported (Branca et al., 2019; Chrobak et al., 2021); however, to our knowledge, data about different HS components are not available representing the first study considering purified lignanamides. The oxidative metabolism of neuronal cells generates large amounts of ROS that are regulated by an elaborate antioxidant system composed of several enzymes, including superoxide dismutase (SOD), catalase, and peroxidases (Massaad and Klann, 2011; Salim, 2017). Beyond the autophagic process, literature data has reported that DMSO toxicity is also related to the generation of ROS (Ock and Rho, 2011; Sumida et al., 2011; Yuan et al., 2014). In agreement with these notions, in our study, we found an increase in NO generation in cells exposed to DMSO, which could inhibit neuronal viability. In accordance with our data, Yuan C. et al. demonstrated that DMSO increases ROS production in a dose-dependent manner in astrocytes cultures (Yuan et al., 2014). It is interesting to notice that LnHS administration counteracted DMSO effects in the co-treatment condition, strongly suggesting that LnHS can enhance neuronal survival, at least in part, by reducing of oxidative stress. In line with our hypothesis, Xu P. W. et al. demonstrated that pretreatment of HUVEC cells with HS polyphenols substantially decreased ROS levels induced by H_2_O_2_ administration (Xu et al., 2023). SIRT3 and SOD2 are key proteins in the oxidative metabolism (Bause and Haigis, 2013; Flynn and Melov, 2013). In the present study, we demonstrated that DMSO-induced oxidative stress is accompanied by the inhibition of SIRT3 and SOD2 and that LnHS prevented the downregulation of both enzymes induced by DMSO. Previously, a modulation of SIRT proteins had been highlighted but only by phytocannabinoids and never by the lignamide fraction (Gerasymchuck et al., 2022; Wang et al., 2022). Similarly, the effects of hemp extracts on the modulation of IL-8 have been found for phytocannabinoids but not considering different extracts and cellular models such as lung epithelial cells, macrophages, and human keratinocytes (Anil et al., 2021; Suryavanshi et al., 2022; Tortolani et al., 2023). Regarding inflammatory cytokine, IL-6 appeared to be increased by DMSO and decreased by the cotreatment with LnHS while TNFα did not significantly change. The two cytokines are potent mediators of neuronal inflammation, which can result in neural injury and ultimately cell death (Li et al., 2014). LnHS action toward the control of IL-6 induction further support its effectiveness against DMSO-induced toxicity; in support of our observation, we and others previous reported that lignamides’ effects on neuronal cells pass also through the modulation of IL -6 and TNF-α (Zhou Y. et al., 2018; Nigro E. et al., 2020).

To our knowledge, this is the first study showing that DMSO inhibits the colony-forming ability of SH-SY5Y neuronal cells. In support of our findings, inhibitory effects following exposure to 1% DMSO on the ability to colony formation ability have been reported on CL1–5 lung cells (Wang C.C. et al., 2012). In our previous study, we observed that LnHS has a marked impact on the colony forming capabilities of U-87 cells, whereas in this study, LnHS did not significantly affect the colony-forming ability of SH-SY5Y cells (data not shown), highlighting a different effect of this compound on different types of cells of the nervous system. Interestingly, we found that LnHS co-administration effectively restores the number of colonies of DMSO-exposed cells.

Our study has points of weakness and strength; limitations are represented by the absence of *in vivo* data that will be performed in future and by the evidence produced on only one neuronal cell line. Furthermore, our data show a beneficial effect of DMSO on neuronal cells but detrimental on glial cells making LnHS as a promising but not definitive pharmacological molecule. Therefore, we believe that our observations need to be confirmed on both primary cells and *in vivo* to suggest the clinical use of LnHS as a neuroprotective drug. Furthermore, since caution should be recommended for the *in vivo* application of LnHS, the improvement of the composition of the components of the lignanamide extract might optimize the beneficial effects and to reduce side effects in future studies.

The LnHS reduction of the DMSO toxic effects, preserving neuronal cells health, and decreasing markers of oxidative stress supports LnHS as a potential feasible compound to support neuronal health.

## 5 Conclusion

Collectively, our results show LnHS counteracts the cytotoxic effects induced by DMSO on SH-SY5Y neuronal cells by improving cell survival through the reduction of autophagic cell death and oxidative stress. This study demonstrated that LnHS administration can protect neuronal cells by mitigating DMSO-induced growth inhibition and oxidative stress. Further studies are needed to clarify the molecular mechanisms underlying the LnHS biological activities on neuronal cells but our data represent a first step toward the identification of HS as an important source of molecules bearing safe protective effects against DMSO in the central nervous system. The combination of DMSO and LnHS effectively slows and prevents DMSO-induced toxicity, indicating that LnHS extracted from HS could represent a suitable strategy to support neuronal health.

## Data Availability

The raw data supporting the conclusion of this article will be made available by the authors, without undue reservation.
