# Supplementary material for: Exploring the neuroprotective activity of a lignanamides-rich extract in human neuroblastoma SH-SY5Y cells under dimethyl sulfoxide-induced stress
Source: Front Cell Dev Biol. 2024 Mar 13;12:1374626. doi: 10.3389/fcell.2024.1374626 (PMC10965797; doi:10.3389/fcell.2024.1374626)
Supplement: Supplementary file 1 [file DataSheet1.docx]

**Exploring the neuroprotective activity of a lignanamides-rich extract in human neuroblastoma SH-SY5Y cells under dimethyl sulfoxide-induced stress**

*Supplementary materials*

**Marta Mallardo** ^1,2^

**Severina Pacifico** ^1^

**Simona Piccolella** ^1^

**Irene Di Meo^3^**

**Maria Rosaria Rizzo**^3^

**Aurora Daniele ^4*^**

**Ersilia Nigro** ^1,2^

^1^ Dipartimento di Scienze e Tecnologie Ambientali, Biologiche, Farmaceutiche, Università della Campania "Luigi Vanvitelli", Via Vivaldi 43, 81100 Caserta, Italy.

^2^ CEINGE, Biotecnologie Avanzate Scarl, Via Gaetano Salvatore 486, 80145 Naples, Italy.

^3^ Dipartimento di Scienze Mediche e Chirurgiche Avanzate, Università della Campania "Luigi Vanvitelli", Napoli, Italy.

^4^ Dipartimento di Medicina Molecolare e Biotecnologie Mediche, Università degli Studi di Napoli "Federico II", 80131 Naples, Italy.

*Corresponding author: aurora.daniele@unina.it

**Figure S1. Structures of the flavonol glycosides found in LnHS**

**Figure S2. Structures and chemical formula of the phenylamides found in LnHS**

**Table S1. Lignanamide constituents detected in LnHS**

| **Name** | **Formula** | **[M-H]^-^ ion (*m/z*)** |
| --- | --- | --- |
| Cannabisin A | C_34_H_30_N_2_O_8_ | 593.1929 |
| Cannabisin B  Cannabisin B isomer | C_34_H_32_N_2_O_8_ | 595.2086 |
| Cannabisin C  Cannabisin C isomer | C_35_H_34_N_2_O_8_ | 609.2242 |
| Cannabisin D  Cannabisin D isomer | C_36_H_36_N_2_O_8_ | 623.2399 |
| Cannabisin E  Cannabisin E isomer | C_36_H_38_N_2_O_9_ | 641.2505 |
| Cannabisin F | C_36_H_36_N_2_O_8_ | 623.2399 |
| Cannabisin H isomer 1  Cannabisin H isomer 2 | C_28_H_31_NO_8_ | 508.1977 |
| Cannabisin I | C_26_H_19_NO_7_ | 456.1089 |
| *N*-caffeoyltyramine dimer  (Cannabisin M, Q) | C_34_H_32_N_2_O_8_ | 595.2086 |
| Grossamide | C_36_H_36_N_2_O_8_ | 623.2399 |
| Demethylgrossamide | C_35_H_34_N_2_O_8_ | 609.2242 |
| 3,3’-didemethylgrossamide | C_34_H_32_N_2_O_8_ | 595.2086 |
| Grossamide K | C_28_H_29_NO_7_ | 490.1871 |
| 3,3’-demethyl-heliotropamide | C_34_H_32_N_2_O_8_ | 595.2086 |
| *N*-caffeoyltyramine/  *N*-feruloyltyramine dimer | C_35_H_34_N_2_O_8_ | 609.2242 |
| *N*-caffeoyltyramine/  *N*-caffeoyloctopamine dimer | C_34_H_32_N_2_O_9_ | 611.2035 |
| *N*-caffeoyltyramine dimer hydroxy derivative | C_34_H_34_N_2_O_9_ | 613.2192 |

**
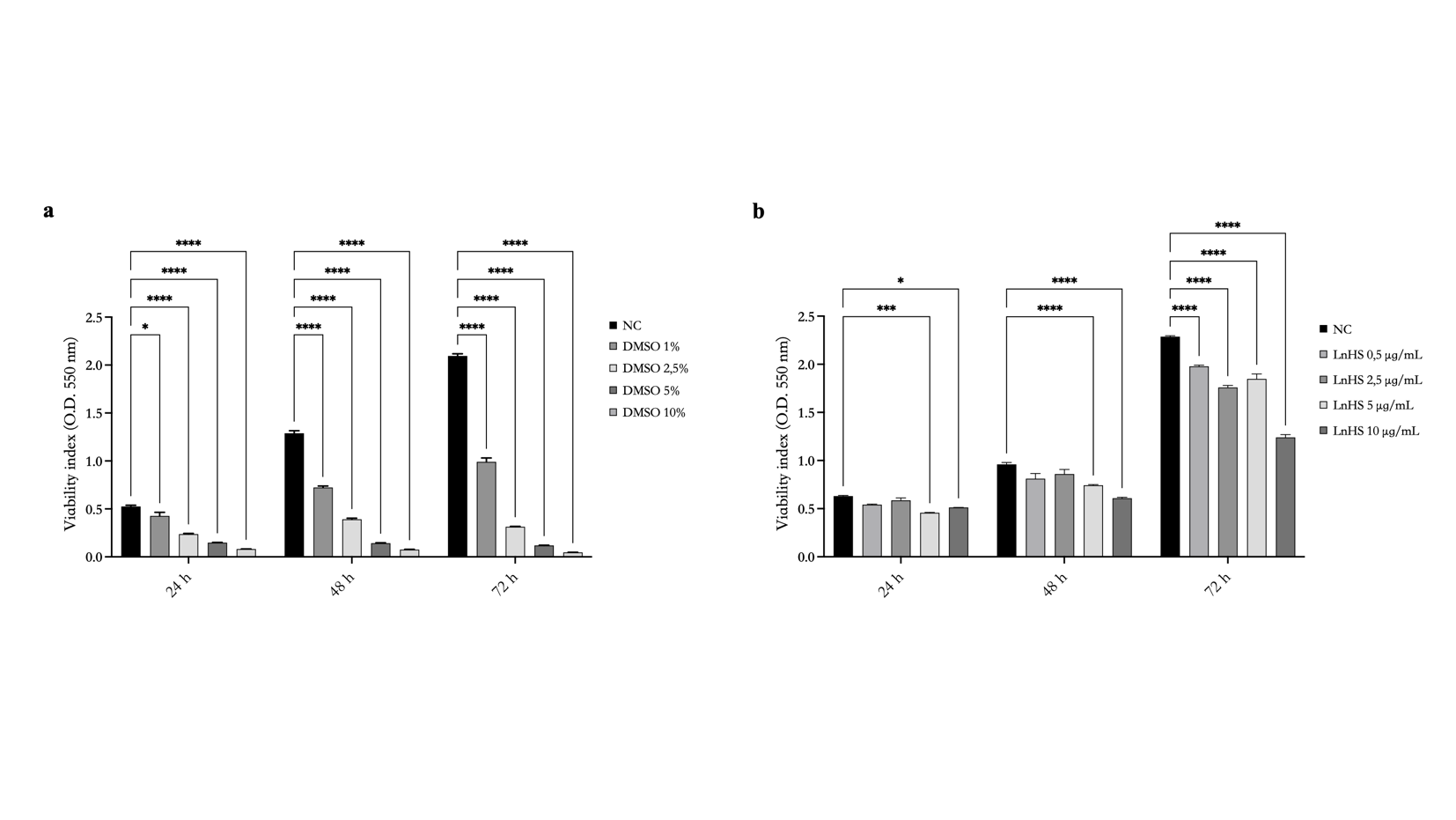
**

**Figure S3. Effects of DMSO and LnHS on the viability of U-87 cells.** Cell viability was assessed using MTT assay after exposure to a) DMSO (1%, 2.5%, 5%, and 10% v/v) and b) LnHS (0.5, 2.5, 5, and 10 μg/mL) for 24, 48, and 72 h. Values are expressed as the mean of two different experiments ± standard error of the mean (SEM). The statistical analysis was evaluated using the two-way ANOVA test. * p value < 0.05; *** p value < 0.001; **** p value < 0.0001 *vs* NC.
